# Women’s experiences of trauma-informed care in midwifery and maternity services and implications for maternal wellbeing: A qualitative systematic review

**DOI:** 10.18332/ejm/225227

**Published:** 2026-10-15

**Authors:** Sofia Thomaidi, Antigoni Sarantaki, Kleanthi Gourounti, Dimitrios Papoutsis, Eirini Orovou

**Affiliations:** 1 Midwifery Department, University of Western Macedonia, Ptolemaida, Greece; 2 Midwifery Department, Faculty of Health and Care Sciences, University of West Attica, Athens, Greece

**Keywords:** trauma-informed care, maternity care, midwifery, women’s experiences, qualitative systematic review

## Abstract

**INTRODUCTION:**

Trauma-informed care has been increasingly advocated within maternity services as an approach to promote safety, autonomy, and respectful care. However, qualitative evidence exploring how participants experienced trauma-informed care across maternity and midwifery services has not been systematically synthesized. This review therefore aimed to synthesize qualitative evidence on women’s experiences of trauma-informed care across maternity services, including midwifery care, during pregnancy, childbirth, and the postpartum period.

**METHODS:**

A systematic review was conducted in accordance with PRISMA 2020 and ENTREQ guidelines. Five electronic databases (PubMed, Scopus, Web of Science, CINAHL, and PsycINFO) were searched for peer-reviewed qualitative studies published in English. Eligible studies explored women’s experiences of trauma-informed, trauma-sensitive, or related care practices within maternity settings. Data were synthesized using thematic synthesis, and methodological quality was appraised using the CASP qualitative checklist.

**RESULTS:**

Fifteen qualitative studies were included. Four analytical themes were identified: 1) creating safety and preventing re-traumatization; 2) respectful communication and informed consent; 3) emotional validation, trust, and relational attunement; and 4) power, control, and women’s agency in maternity care. Across studies, trauma-informed care was experienced not as a discrete intervention but as a relational approach embedded in everyday maternity practices.

**CONCLUSIONS:**

Women’s experiences suggest that trauma-informed principles may contribute to positive care experiences and support psychological safety, autonomy, and relational wellbeing across the perinatal period, when embedded within routine midwifery and maternity practice.

## INTRODUCTION

Pregnancy, childbirth, and the postpartum period represent significant life transitions that can be experienced as empowering and meaningful, but also as physically and psychologically demanding^1^. For many women, interactions with maternity services shape not only clinical outcomes but also emotional well-being, sense of autonomy, and long-term perceptions of motherhood. The quality of care received during this period has been shown to influence maternal satisfaction, engagement with health services, and psychological adjustment following birth^2^.

A substantial proportion of women enter pregnancy with a history of trauma, including exposure to adverse childhood experiences, sexual abuse, intimate partner violence, forced medical procedures, or previous traumatic birth experiences^1^. International studies estimate that approximately 40–60% of women of reproductive age report exposure to at least one traumatic event during their lifetime^3^. In addition to interpersonal trauma, trauma-informed frameworks increasingly recognize the impact of structural and racial trauma, including experiences of racism, discrimination, and systemic inequities within healthcare systems. These experiences may shape how individuals engage with maternity services and can contribute to heightened vulnerability, mistrust, and disparities in maternal health outcomes. Such exposures are highly prevalent among women of reproductive age worldwide and are strongly associated with increased vulnerability during the perinatal period^1-3^. Research has consistently indicated links between trauma histories and heightened risk of perinatal anxiety, depression, fear of childbirth, and birth-related post-traumatic stress symptoms. Importantly, trauma does not affect only a small or marginalized subgroup of people entering pregnancy and childbirth; rather, it represents a widespread and often hidden determinant of maternal health^3^.

Maternity care encounters may unintentionally re-activate traumatic memories or emotional responses. Invasive examinations, exposure of the body, loss of privacy, lack of informed consent, dismissive communication, or experiences of power imbalance can be perceived as threatening or overwhelming, particularly for women with prior trauma^4^. Such experiences may contribute to feelings of loss of control, fear, and distress, and in some cases lead to avoidance of maternity services or disengagement from postnatal care^3^. The concept of obstetric violence and reports of disrespectful or abusive maternity care have further highlighted the potential for harm when women’s emotional safety and autonomy are not adequately protected^5^.

In response to growing recognition of these issues, trauma-informed care (TIC) has emerged as an important framework within health services^5^. TIC is grounded in an understanding of the pervasive impact of trauma and seeks to create environments and practices that promote psychological safety, trust, and empowerment. Core principles of TIC include safety, trustworthiness, choice, collaboration, and empowerment, alongside a commitment to recognizing and responding to trauma without requiring disclosure^6^. Rather than focusing solely on the treatment of trauma-related symptoms, TIC emphasizes the prevention of re-traumatization and the promotion of supportive, respectful care for all service users^6^.

Within maternity and midwifery services, trauma-informed approaches align closely with the philosophy of woman-centered and respectful maternity care^4^. These approaches emphasize relational continuity, shared decision-making, informed consent and recognition of individuals receiving maternity care as active participants in their care. International organizations, including the World Health Organization (WHO), have highlighted the importance of respectful and dignified maternity care as a fundamental component of quality maternal health services^7^. Trauma-informed principles provide a complementary lens through which respectful care can be operationalized, particularly for women with complex psychosocial needs^4,8^.

Despite increasing policy and professional interest in trauma-informed maternity care, implementation remains inconsistent across settings. One challenge lies in the complexity of TIC as a relational, context-dependent practice rather than a discrete clinical intervention^4^. As a result, traditional quantitative approaches have struggled to capture the nuanced ways in which trauma-informed principles are enacted and experienced in everyday maternity care. While some quantitative studies have examined associations between trauma-informed approaches and selected maternal outcomes, robust evidence from randomized or controlled designs remains limited^9^.

Qualitative research has therefore played a central role in advancing understanding of TIC within maternity contexts^10,11^. Through in-depth interviews, focus groups and narrative accounts, qualitative studies have explored how women perceive safety, trust, control and support during pregnancy and childbirth^10^. Such studies provide rich insights into women’s lived experiences, including the ways in which interactions with midwives and other maternity care providers can either mitigate or exacerbate distress. Women’s voices are particularly important in this field, as TIC is fundamentally concerned with how care is experienced rather than solely with how it is delivered.

However, the existing qualitative literature is fragmented, with studies conducted across diverse settings, populations, and health systems. Some research focuses on women with specific trauma histories, such as survivors of sexual violence or intimate partner violence, while other studies examine broader experiences of maternity care among women who may or may not identify as having experienced trauma^12^. Moreover, TIC is variably defined and operationalized across studies, complicating efforts to draw overarching conclusions. To date, qualitative evidence exploring women’s experiences of TIC in midwifery and maternity services has not been systematically synthesized in a focused and comprehensive manner^11,13^.

Previous reviews in related areas have tended to address perinatal trauma more broadly, to prioritize professional or organizational perspectives, or to combine qualitative and quantitative evidence without foregrounding women’s lived experiences^13^. As a result, there remains a need for a qualitative systematic review that centers women’s voices and examines how TIC is understood, perceived and experienced within maternity services. Synthesizing this evidence is essential for identifying common themes, highlighting facilitators and barriers to trauma-informed practice from women’s perspectives, and informing the development of maternity services that are responsive to women’s psychological and emotional needs.

Although qualitative studies have explored trauma-informed care across diverse populations and maternity care contexts, including women affected by interpersonal trauma, migration, marginalization, and other vulnerabilities, the evidence remains fragmented and lacks a focused synthesis of women’s experiences^14^ . Trauma-informed maternity care has also been described as a relational and context-dependent approach influenced by organizational structures, care practices, and interactions with maternity professionals^8,15^.

This systematic review aims to synthesize existing qualitative evidence on women’s experiences of trauma-informed care within midwifery and maternity services during pregnancy, childbirth, and the postpartum period. Specifically, the review explores how women perceive and experience trauma-informed care across the perinatal continuum and identifies aspects of maternity care that facilitate or hinder trauma-informed experiences.

## METHODS

### Design

This systematic review was conducted in accordance with the Preferred Reporting Items for Systematic Reviews and Meta-Analyses (PRISMA) 2020 guidelines^16^ and the Enhancing Transparency in Reporting the Synthesis of Qualitative Research (ENTREQ) statement^17^. A qualitative evidence synthesis approach was selected to enable in-depth exploration and integration of women’s lived experiences of TIC across diverse maternity contexts. This approach prioritizes meaning, interpretation, and experiential understanding rather than statistical aggregation. This review was registered in PROSPERO (CRD420261280530) on 7 January 2026.

### Eligibility criteria

Eligibility criteria were defined *a priori* using the Population–Phenomenon of Interest–Context (PICo) framework, which is appropriate for qualitative evidence synthesis.

*Population*

Studies were eligible if they included women aged ≥18 years who were pregnant, giving birth, or in the postpartum period. Studies focusing on women with or without a disclosed history of trauma were included. Studies that exclusively examined healthcare professionals’ perspectives were excluded.

*Phenomenon of interest*

The phenomenon of interest was women’s experiences, perceptions or views of TIC, trauma-informed approaches or trauma-sensitive practices within maternity services. Studies addressing experiences of safety, autonomy, trust, communication, consent, or re-traumatization in maternity care were considered relevant, even if TIC was not explicitly labeled as such.

*Context*

Studies conducted in midwifery-led or maternity care settings, including antenatal, intrapartum and postnatal services, were eligible. No restrictions were placed on geographical location or healthcare system.

### Types of studies

Qualitative primary research studies employing methods such as interviews, focus groups, ethnography, or qualitative components of mixed-methods studies were included, provided that qualitative findings were reported separately. Quantitative-only studies, editorials, commentaries, protocols, dissertations, and conference abstracts were excluded.

### Language and publication status

Only studies published in English in peer-reviewed journals were included. No restrictions were applied based on year of publication.

### Search strategy

A comprehensive and systematic search strategy was developed in consultation with established guidance for qualitative systematic reviews. Electronic database searches were conducted in PubMed, Scopus, Web of Science, CINAHL, and PsycINFO on 15 January 2026, which represented the final search date. Given the evolving conceptualization of TIC within maternity services, no temporal limits were applied during the search to avoid the exclusion of relevant foundational qualitative studies. The full search algorithms used in each database are presented in Supplementary file Table 1.

### Study selection

All records were imported into reference management software, and duplicates were removed prior to screening. Titles and abstracts were screened against the eligibility criteria, followed by full-text assessment of potentially relevant articles. Two reviewers independently screened titles and abstracts against the eligibility criteria. Full-text articles were subsequently assessed for inclusion. Any disagreements were resolved through discussion and, where necessary, consultation with a third reviewer.

### Data extraction

Data were extracted independently by two reviewers using a predefined data extraction approach developed for the purposes of this review. Extracted information included author, year of publication, country, participant characteristics, trauma context, data collection methods, analytical approach, and key qualitative findings relevant to women’s experiences of trauma-informed care. Following independent extraction, the reviewers compared the extracted data, and any discrepancies were resolved through discussion and consensus.

### Quality appraisal

The methodological quality of included studies was appraised using the Critical Appraisal Skills Program (CASP) qualitative checklist^18^. A qualitative methodology was appropriate given the focus on women’s lived experiences and perceptions of TIC within maternity services. Quality appraisal was conducted to enhance transparency and interpretive depth; no studies were excluded based on methodological quality, and no numerical scoring was applied. The CASP tool comprises ten items assessing clarity of research aims, appropriateness of qualitative methodology, recruitment strategy, data collection, researcher–participant relationship (reflexivity), ethical considerations, rigor of data analysis, clarity of findings, and overall value of the research.

Quality appraisal was undertaken to enhance transparency and interpretive depth rather than to exclude studies based on quality alone. All included studies were considered to provide sufficiently rich qualitative data to contribute meaningfully to the synthesis. Variations in methodological reporting were noted and considered during the interpretation of findings. In addition, recruitment strategies were generally appropriate to the study aims, enabling inclusion of women with relevant lived experiences of maternity care.

### Data synthesis

A thematic synthesis approach, as described by Thomas and Harden^19^, was used to integrate findings across the included studies. This method was selected to allow for systematic comparison and interpretation of qualitative evidence derived from diverse maternity care contexts while preserving women’s lived experiences.

Synthesis proceeded through three iterative stages: 1) line-by-line coding of extracted findings relevant to women’s experiences of TIC; 2) development of descriptive themes by grouping conceptually similar codes across studies; and 3) generation of analytical themes that captured higher order patterns and shared meanings across the dataset. Only data derived directly from women’s accounts were coded and synthesized. Where studies included additional participant groups (e.g. healthcare professionals), these data were used solely to contextualize women’s experiences and were not included in the analytic synthesis. Throughout the process, attention was paid to how women described safety, trust, autonomy, communication, validation, and experiences of re-traumatization or support within maternity care. Two reviewers independently participated in the coding and development of themes. Discrepancies in coding or interpretation were discussed and resolved through consensus among the research team. The synthesis aimed to move beyond individual study findings to produce an integrated, interpretive understanding of women’s experiences of TIC in midwifery and maternity services.

## RESULTS

### Study characteristics

A total of 162 records were identified through database searching, including 46 from PubMed, 12 from Scopus, 52 from EBSCO, 30 from Web of Science, and 22 from PsycINFO. After completion of the screening and eligibility assessment process, 15 studies met the inclusion criteria and were included in the qualitative synthesis. The study selection process is illustrated in the PRISMA flow diagram (Figure 1).

**Figure 1:**
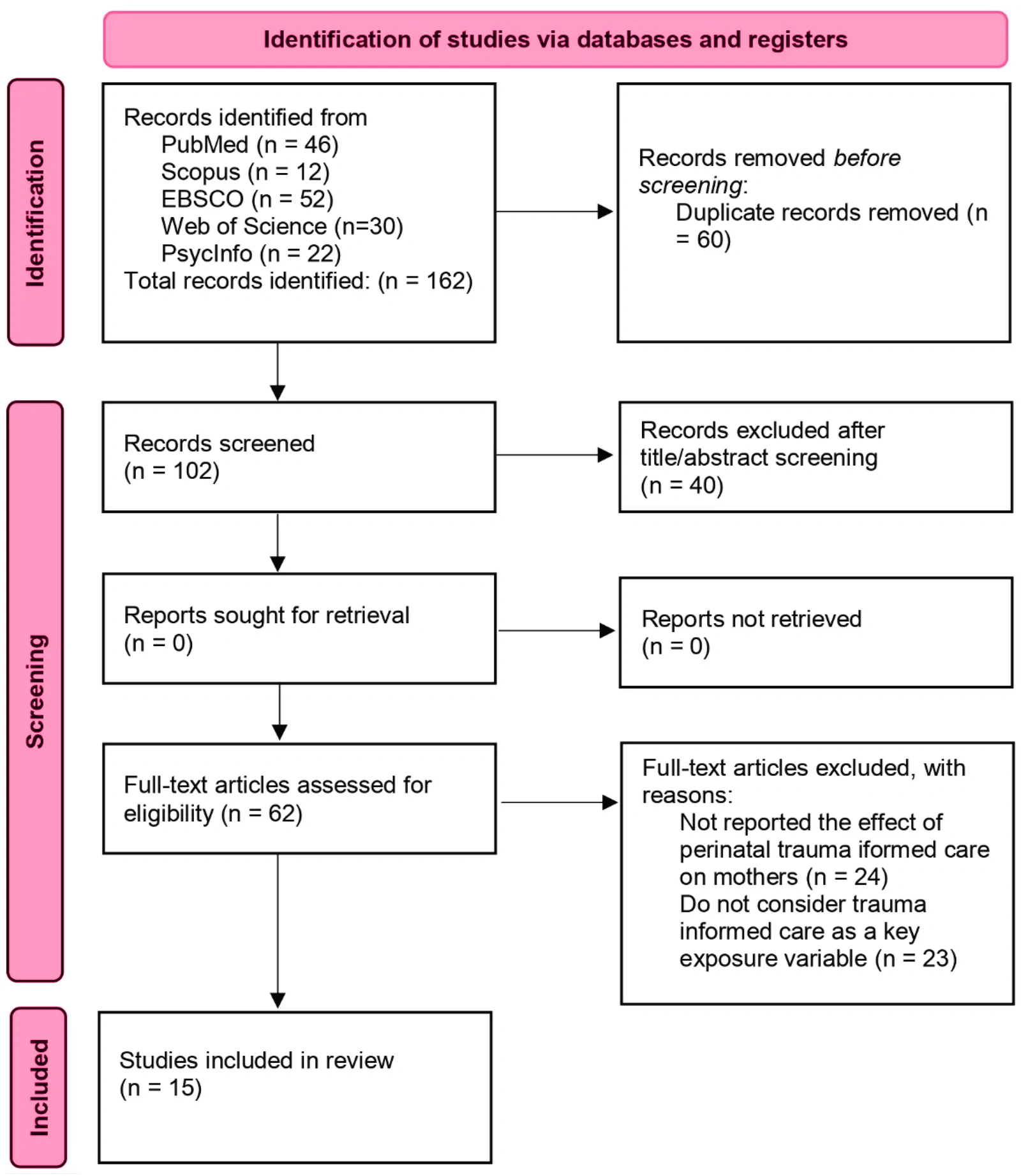
Flow chart of the included studies

The studies were published between 2017 and 2025 and were conducted across a range of high-income countries, including the United Kingdom, the United States, Canada, Ireland, and Australia. All included studies employed qualitative or mixed-methods designs with a clearly identifiable qualitative component capturing women’s experiences of maternity or midwifery care in the context of trauma. The populations studied consisted exclusively of adult women, including pregnant women, postpartum women, and women reflecting retrospectively on their maternity care experiences. Trauma contexts varied across studies and are summarized in Table 1. Several studies focused on women with multiple or cumulative trauma exposures^20-24^. Several studies focused on women with multiple or cumulative trauma exposures^25,26^.

**Table 1:** Characteristics of the included qualitative studies examining women’s experiences of trauma-informed maternity care, including author, year, country, sample size, study design, data collection methods, trauma context and key experiential themes

*Author* *Year* *Country*	*Participants*	*Data collection method*	*Data collection period*	*Analytical approach*	*Trauma context*	*Key experiential themes*
Searle et al.^20^ 2017 Canada	13 queer-identified women with perinatal care experiences	Semi-structured interviews	Not reported	Phenomenological thematic analysis	Structural marginalization	Exclusion, invisibility, validation
Burrow et al.^27^ 2018 Canada	13 queer women with perinatal care experiences	In-depth interviews	2015–2017	Phenomenological/thematic analysis	Structural oppression	Vulnerability, harm, compromised autonomy
Sobel et al.^21^ 2018 USA	30 women up to ~3 years after childbirth	Semi-structured interviews	Not reported	Grounded theory	Sexual trauma	Control, consent, communication
Gokhale et al.^25^ 2020 USA	30 pregnant women	Semi-structured interviews	Not reported	Thematic analysis	Multiple lifetime trauma	Safety, trust, validation
Olsen et al.^28^ 2021 USA	154 women with history of one or more pregnancies	Open-ended survey responses	Not reported	Descriptive qualitative analysis	Adverse childhood experiences	Preferences for screening
Perera et al.^29^ 2023 Canada	9 perinatal women	In-depth interviews	Not reported	Constructivist grounded theory	Interpersonal trauma	Safety, unmet needs
Tsakmakis et al.^22^ 2023 Australia	24 perinatal women	Online interviews	Not reported	Feminist thematic analysis	Birth trauma	Loss of control, power imbalance
Yount-Tavener and Fay^30^ 2023 USA	8 women	Open-ended online survey	Not reported	Thematic analysis	Intrapartum transfer	Disruption, loss of control
Montgomery and Chang^31^ 2023 UK	8 perinatal women	Interviews/focus groups	Not reported	Content analysis	Childhood sexual abuse	Emotional safety, communication
Toke et al.^23^ 2024 Australia	7 refugee women	Semi-structured interviews	Not reported	Reflexive thematic analysis	Refugee trauma	Access, interpreting, control
Collins and Skarparis^32^ 2024 UK	5 trafficked pregnant women	Semi-structured interviews	Not reported	Thematic analysis identifying five key themes	Trafficking	Lack of individualized care
Jackson et al.^24^ 2024 Canada	6 pregnant women	Arts-based narratives	Not reported	Interpretive analysis	Intimate partner violence	Triggers, resilience
Renbarger et al.^33^ 2024 USA	16 perinatal women	Semi-structured interviews	Not reported	Content analysis	Substance use disorder	Barriers, judgement
Itani et al.^26^ 2025 USA	27 pregnant women	Interview-administered structured interviews	Not reported	Inductive thematic analysis	Diverse trauma exposures	Agency, responsiveness
Cull et al.^15^ 2025 UK	4 women	Semi-structured interviews	Not reported	Reflexive thematic analysis	Previous traumatic birth	Timing, sensitive dialogue

‘Trauma context’ refers to the type of trauma or source of distress described by the original study authors in relation to the study population. This classification reflects the context of the included studies and does not imply individual diagnoses, trauma severity, or measured exposure.

Data collection methods predominantly involved in-depth semi-structured interviews, although some studies also used focus groups, arts-based approaches, or open-ended qualitative survey responses. Analytical approaches included thematic analysis, reflexive thematic analysis, qualitative content analysis, and interpretative phenomenological analysis. Where studies included additional participant groups (e.g. healthcare professionals), only findings derived from women’s accounts were extracted and synthesized, with professional perspectives used solely to contextualize women’s experiences. Across studies, TIC was not always explicitly labeled as such; however, women consistently described care experiences in relation to core trauma-informed principles, including emotional and physical safety, trust, respectful communication, autonomy, informed consent, validation, and avoidance of re-traumatization. These shared experiential dimensions formed the basis for the subsequent thematic synthesis^15,20-27,29,31-33^. Table 2 provides an overview of the characteristics, trauma contexts, and reported outcomes of the included studies.

**Table 2:** Overview of trauma context, exposure to TIC, women’s reported experiences, qualitative outcomes, and implications for maternal wellbeing across the included qualitative studies

*Author* *Year* *Country*	*Trauma context*	*Exposure to trauma-informed care*	*Reported experiences*	*Primary qualitative outcomes*	*Implications for maternal wellbeing/care quality*
Searle et al.^20^ 2017 Canada	Structural marginalization	Perinatal TIC	Validation of identity improved experiences	Emotional safety, trust	Reduced alienation, improved relational care
Burrow et al.^27^ 2018 Canada	Structural oppression	Perinatal TIC	Harmful interactions reported	Psychological distress, loss of agency	Compromised wellbeing, need for inclusive care
Sobel et al.^21^ 2018 USA	Sexual trauma	Prenatal TIC	Preference for control and consent	Autonomy, psychological safety	Reduced distress during care encounters
Gokhale et al.^25^ 2020 USA	Multiple trauma	Prenatal TIC	Empathic communication valued	Trust, emotional safety	Increased engagement with care
Olsen et al.^28^ 2021 USA	Childhood adversity	Prenatal TIC	Support for sensitive screening	Emotional safety, acceptability	Improved screening experiences
Perera et al.^29^ 2023 Canada	Interpersonal trauma	Perinatal TIC	Mixed care experiences	Emotional validation, trust	Support for psychological wellbeing
Tsakmakis et al.^22^ 2023 Australia	Birth trauma	Perinatal TIC	Loss of control reported	Reduced agency, distress	Impacts on emotional adjustment
Yount-Tavener and Fay^30^ 2023 USA	Care transfer	Intrapartum TIC	Disrupted birth expectations	Emotional distress	Need for postnatal processing
Montgomery and Chang^31^ 2023 UK	Childhood sexual abuse	Perinatal TIC	Need for sensitive communication	Emotional safety, trust	Reduced re-traumatization
Toke et al.^23^ 2024 Australia	Refugee trauma	Antenatal TIC	Importance of safe interpreting	Participation, agency	Improved access and involvement
Collins and Skarparis^32^ 2024 UK	Trafficking	Perinatal TIC	Lack of individualized care	Disempowerment, distress	Need for continuity of care
Jackson et al.^24^ 2024 Canada	Intimate partner violence during pregnancy	Antenatal TIC	Trigger awareness and coping	Emotional regulation, resilience	Supportive care environment
Renbarger et al.^33^ 2024 USA	Substance use disorder	Perinatal TIC	Non-judgemental care desired	Engagement, trust	Improved service utilization
Itani et al.^26^ 2025 USA	Diverse trauma	Prenatal TIC	Preference for responsive providers	Autonomy, emotional safety	Enhanced care satisfaction
Cull et al.^15^ 2025 UK	Traumatic birth	Perinatal TIC	Importance of timing and dialogue	Psychological safety, trust	Improved care interactions

‘Trauma context’ is used analytically to describe the contextual conditions shaping women’s experiences of maternity care and the qualitative outcomes identified in each study. It reflects experiential and relational contexts rather than clinical or diagnostic categories. Antenatal TIC: trauma-informed care delivered during pregnancy. Intrapartum TIC: trauma-informed care delivered during labor and childbirth. Perinatal TIC: trauma-informed care spanning pregnancy, childbirth and the postpartum period. Searle et al.^20^ and Burrow et al.^27^ draw on the same sample of 13 queer-identified women in rural Nova Scotia, but present different analytical perspectives on participants’ experiences. Both studies were included because they contribute distinct qualitative insights relevant to trauma-informed maternity care.

### Methodological quality assessment

The methodological quality of the included studies was assessed using the CASP Qualitative Checklist. Overall, the studies demonstrated satisfactory methodological quality. Most studies clearly stated their aims, employed appropriate qualitative methodologies and research designs, and provided adequate descriptions of recruitment, data collection, and findings. Ethical considerations were generally well addressed. The criterion least consistently reported was researcher reflexivity and consideration of the researcher–participant relationship. Variability was also observed in the depth of reporting of analytical procedures. Nevertheless, all studies were considered sufficiently robust to contribute meaningful qualitative evidence to the synthesis. A summary of the methodological quality assessment is presented in Supplementary file Table 2.

### Thematic synthesis

The thematic synthesis generated four overarching analytical themes that captured shared patterns in women’s experiences of TIC across maternity and midwifery services. These themes reflect how women described safety, communication, power, and emotional validation within care encounters, particularly in the context of prior trauma.

### Theme 1: Creating safety and preventing re-traumatization

This theme was identified across seven studies^15,21,24,25,27,29,31^ and reflected women’s experiences of physical and emotional safety within maternity care. Across studies, women consistently emphasized the importance of physical and emotional safety within maternity care^21,25,29,31^. Trauma-informed care was experienced positively when healthcare environments, clinical interactions, and procedures minimized the risk of re-traumatization. Women described feeling unsafe when care was rushed, invasive, or poorly explained, particularly during intimate examinations or labor interventions. Women reported feeling unsafe when care was rushed, invasive, or inadequately explained, especially in the context of previous sexual abuse, intimate partner violence, or cumulative trauma histories^15,27,31^. Conversely, practices such as respectful pacing, attention to personal boundaries, and provider awareness of trauma histories contributed to feelings of security and trust. For many women, perceived safety was a prerequisite for engaging with maternity services and maintaining continuity of care^24^. Women frequently described the importance of feeling emotionally and physically safe within maternity care encounters. A supportive and trusting relationship with care providers was often perceived as essential for mitigating distress and facilitating engagement with care. As one participant described^25^:

*‘The only way my health care providers can help me with my trauma is every time I come, make me feel like I’m at home. Make me feel comfortable. Make me feel safe and make me feel like I have nothing to worry about.’*

## Theme 2: Respectful communication and informed consent

This theme was identified across six studies^15,21,22,25,29,31^ and highlighted the importance of clear communication, informed consent, and collaborative decision-making in trauma-informed maternity care. Clear, respectful, and transparent communication emerged as a central component of trauma-informed maternity care. Women valued healthcare professionals who explained procedures in advance, sought explicit consent, and provided opportunities to ask questions or decline interventions^21,25,29^. In contrast, women described distress when procedures were undertaken without adequate explanation or when consent was assumed rather than actively obtained. Lack of communication was frequently linked to feelings of powerlessness, fear, and loss of bodily autonomy^22,31^. Trauma-informed communication was therefore understood not merely as information provision, but as an ongoing, relational process that supported women’s sense of agency throughout pregnancy, birth, and postpartum care^15^. Clear and respectful communication was frequently described as essential for supporting women’s sense of autonomy and control during maternity care. Women valued care providers who explained options and actively involved them in decision-making. As one participant explained^21^:

*‘My primary doctor, he was just like, “What do you want? Do you want to do C-section? Do you want to do vaginal delivery? You let me know and we’ll go that route as long as it is safe”. That’s what I wanted to hear.’*

### Theme 3: Validation, trust, and emotional attunement

This theme was identified across seven studies^15,20,25-27,29,31^ and reflected women’s experiences of emotional validation, trust, and attuned relationships with maternity care providers. Across studies, emotional validation and attuned care emerged as central relational outcomes, closely associated with trust in care providers and women’s perceived emotional safety within maternity services. For example, women highlighted the significance of being believed, heard, and emotionally validated by maternity care providers. TIC was characterized by providers’ ability to acknowledge women’s fears, prior experiences, and emotional responses without judgment or dismissal^29,31^. Trust developed when women perceived providers as empathetic, attentive, and responsive to emotional cues^25,26^. Conversely, dismissive or minimizing responses undermined trust and intensified feelings of vulnerability^20,27^. For many women, emotional attunement and validation were as important as clinical competence in shaping their overall care experience^15^. Women frequently described the importance of emotional validation and sensitive responses from care providers when discussing difficult birth experiences or trauma histories. Emotional attunement and acknowledgment of women’s experiences were perceived as essential for building trust within maternity care relationships. As one participant described^29^:

*‘Even right now, it’s seeing scenes of childbirth, like in movies and TV shows, I will do something else when that is playing on the TV. I will completely distract myself and do the dishes, play with my baby, and do a whole bunch of stuff, so I’m not paying attention to that scene. Because I want to cry or scream or just shout, why didn’t I get that? I didn’t get that at all.’*

### Theme 4: Power, control, and women’s agency in care

This theme was identified across seven studies^20-22,25-27,32^ and reflected women’s experiences of power, control, and agency within maternity care encounters. Women’s experiences indicate that agency and shared decision-making constitute critical experiential outcomes of trauma-informed maternity care, influencing women’s engagement, confidence, and sense of control across the perinatal continuum. Experiences of TIC were closely linked to women’s perceptions of power and control within maternity services. Women described positive care encounters when they felt involved in decision-making, respected as knowledgeable about their own bodies, and supported to exercise choice^21,25,26^. In contrast, hierarchical care structures and authoritative decision-making practices often reinforced feelings of disempowerment, particularly for women with prior experiences of trauma or marginalization^20,27,32^. Trauma-informed care was therefore experienced as care that actively redistributed power, enabling women to reclaim agency within maternity and midwifery contexts^22^. Women frequently described experiences of diminished control and disempowerment when maternity care deviated significantly from their expectations or preferences. When decision-making processes were perceived as dominated by clinical authority or institutional constraints, women reported feelings of loss of agency during childbirth. As one participant described^22^ *:*

*‘I felt a bit out of control of the whole thing, was just kind of panicking a little bit in general because it had gone so not to plan, like everything I planned for up until that point I’d not been able to do. Up to that point, it was like, well, what even is the point?’*

## DISCUSSION

This qualitative systematic review synthesized women’s experiences of trauma-informed care within midwifery and maternity services across diverse care settings. The findings suggest that women perceived trauma-informed care as a relational approach embedded within everyday maternity care practices rather than as a discrete intervention. Across studies, experiences of safety, trust, communication, emotional validation, and agency emerged as closely interconnected aspects of positive maternity care encounters. These findings are consistent with previous literature emphasizing the importance of respectful, woman-centered, and trauma-informed approaches in supporting women’s experiences of maternity care. Women’s accounts indicate that TIC is recognized through clinicians’ sensitivity to bodily boundaries, emotional cues, and the potential for distress, without requiring women to explicitly disclose traumatic experiences. Women described feeling supported when care providers acknowledged vulnerability and adapted their communication and practices accordingly, particularly during intimate or potentially triggering procedures^34^. This approach appears to reduce the emotional burden associated with disclosure while promoting psychological safety, trust, and a sense of control within maternity care encounters^29^.

Clear, respectful, and transparent communication emerged as a central component of women’s experiences of trauma-informed maternity care. Women valued care encounters in which procedures were explained in advance, consent was actively sought, and opportunities were provided to ask questions, pause, or decline interventions. Such practices supported women’s sense of control and bodily autonomy, particularly during intimate or invasive aspects of care^21,25,29^. In contrast, assumed consent, limited explanation, or authoritative decision-making were described as distressing and were often associated with fear, loss of control, and feelings of disempowerment. These experiences suggest that respectful communication and ongoing consent operate as relational process outcomes, supporting women’s sense of bodily autonomy and reducing feelings of fear and powerlessness during maternity care.

These findings suggest that consent in maternity care functions not only as an ethical requirement but as a relational process that shapes women’s psychological safety and trust within clinical encounters^35^. These findings are consistent with previous research highlighting the importance of emotional safety, trust, and respectful communication in maternity care. For example, previous studies have emphasized that trauma-informed approaches can reduce the risk of re-traumatization and support women’s psychological wellbeing during the perinatal period. Similar themes have been reported in qualitative and integrative reviews examining re-traumatization and women’s experiences of maternity care, which identify communication, autonomy, and emotional validation as central components of supportive perinatal care^11^.

Women’s experiences underscored the importance of emotional validation and feeling believed by maternity care providers. Trauma-informed care was recognized when providers demonstrated empathy, emotional presence, and responsiveness to women’s expressed concerns, contributing to feelings of safety and trust^11^. Trust developed primarily through emotionally attuned relationships rather than technical competence alone, particularly for women with prior trauma experiences^11^. Dismissive or minimizing responses were described as distressing and were perceived as reinforcing earlier experiences of disempowerment^36^. Across studies, women’s experiences of TIC were shaped by organizational and institutional factors within maternity services. Hierarchical models of care, time pressures, and rigid protocols were perceived as limiting women’s autonomy and opportunities for shared decision-making. Trauma-informed care was experienced more positively when care structures allowed flexibility, continuity, and active involvement in decision-making, supporting women’s sense of agency during pregnancy and birth^11^. Women described these organizational conditions as affecting their experiences of agency, emotional safety, and engagement with maternity care.

This synthesis contributes to the maternity care literature by illustrating that trauma-informed maternity care extends beyond individual clinician skills or discrete training initiatives. Women’s experiences indicate that trauma-informed practices are shaped by organizational cultures, policy frameworks, and professional roles within maternity services. These findings suggest the importance of considering structural and institutional factors when implementing trauma-informed approaches, particularly in relation to continuity of care, decision-making processes, and flexibility within care pathways. Viewing trauma-informed maternity care through a feminist-informed analytical lens offers valuable insights into how care relationships can better support women’s autonomy, dignity, and embodied experiences within routine maternity care.

Taken together, the findings suggest that women perceived trauma-informed maternity care as supporting psychological safety, trust, autonomy, and emotional validation. These experiences appeared to be shaped by both interpersonal relationships and organizational contexts, and may be relevant to the development of more responsive and supportive maternity care services.

### Implications for future practice

The findings of this review have important implications for the implementation of TIC within maternity services. Given their sustained and often continuous contact with women across pregnancy, childbirth, and the postpartum period, midwives are particularly well positioned to operationalize trauma-informed principles in everyday maternity care^8,11^. Practices such as clear and respectful communication, ongoing consent, emotional attunement, and support for women’s agency align closely with the core competencies and relational orientation of midwifery practice. When enacted consistently, these practices are likely to support key care-related outcomes identified in this review, including psychological safety, trust in care providers, women’s sense of autonomy, and sustained engagement with maternity services. Future implementation efforts may benefit from strengthening midwifery-led roles within multidisciplinary maternity teams, particularly in relation to continuity of care, shared decision-making, and advocacy for women’s preferences. Embedding trauma-informed principles within midwifery education, clinical supervision, and reflective practice frameworks may support more consistent and sustainable implementation across care settings. At a service level, implementing trauma-informed principles requires organizational conditions that enable relational care, including adequate time, continuity models, and supportive leadership, rather than reliance on individual clinician skills alone. Taken together, the findings suggest that women perceived trauma-informed maternity care as supporting psychological safety, trust, autonomy, and emotional validation. These experiences appeared to be shaped by both interpersonal relationships and organizational contexts and may be relevant to the development of more responsive maternity care services.

Future research should further explore how trauma-informed principles can be implemented consistently across different maternity care settings and healthcare systems. In particular, longitudinal and implementation-focused studies are needed to examine how trauma-informed approaches influence maternal wellbeing, service engagement, and early relational outcomes such as mother–infant bonding. Additional research in diverse cultural and healthcare contexts, including low- and middle-income countries, would also contribute to a more comprehensive understanding of trauma-informed maternity care and its potential benefits for women and families.

### Strengths and limitations

A key strength of this qualitative systematic review lies in its focus on women’s lived experiences of TIC across the perinatal continuum. By synthesizing qualitative evidence from diverse maternity settings and trauma contexts, the review provides a nuanced understanding of how trauma-informed principles are perceived and enacted in everyday maternity care. The use of systematic and transparent methods, guided by PRISMA and ENTREQ frameworks, enhances the rigor and credibility of the synthesis, while thematic analysis enables the identification of higher order themes relevant across different care models and health systems. By foregrounding women’s experiential accounts, the review also highlights outcome-relevant domains – such as psychological safety, trust, autonomy, and engagement with care – that are often underrepresented in outcome-focused maternity research.

Several limitations should be acknowledged. The review was restricted to studies published in English, which may introduce language bias and limit the inclusion of potentially relevant studies published in other languages. In addition, TIC was not consistently defined across studies, requiring interpretive synthesis of experiences aligned with trauma-informed principles. However, this interpretive approach is consistent with qualitative evidence synthesis methodologies and was applied systematically to enhance analytical transparency. As with all qualitative syntheses, the findings are shaped by the quality and reporting depth of the included studies. Despite these limitations, the review offers robust insights into relational and experiential processes that inform trauma-informed maternity care, with clear relevance for future practice, education, and service development. In addition, the review included only peer-reviewed journal articles and did not incorporate grey literature, which may introduce publication bias. The included studies also represented diverse trauma contexts (e.g. childhood abuse, intimate partner violence, migration-related trauma, and birth trauma), and this heterogeneity may limit the extent to which general conclusions can be drawn. Although methodological quality was assessed using the CASP checklist, no studies were excluded based on quality, meaning that some included studies were small-scale qualitative investigations. Finally, most studies relied on women’s self-reported experiences, which may be influenced by recall bias or social desirability bias.

## CONCLUSIONS

This systematic review demonstrates that women experience trauma-informed maternity care as a relational, embodied, and gendered practice embedded within power-laden healthcare systems. Trauma-informed maternity care is best understood not as a technical intervention alone, but as a relational approach that supports the bodily autonomy of people receiving maternity care, voice, and psychological safety within maternity services. Viewing trauma-informed maternity care through a feminist-informed lens offers valuable insights into how maternity services can better support women’s dignity, agency, and wellbeing across the perinatal continuum.

## Supplementary Material



## Data Availability

The data supporting this research are available from the authors on reasonable request.
